# The Prosocial-Culture-Work Nexus: An Integrative Literature Review and Future Research Agenda

**DOI:** 10.3390/bs13030203

**Published:** 2023-02-25

**Authors:** Stephen Gibb

**Affiliations:** School of Business and Creative Industries, University of the West of Scotland, Paisley PA1 2BE, UK; stephen.gibb@uws.ac.uk

**Keywords:** prosocial, culture, work, organization

## Abstract

Organization culture is a potential antecedent and moderator of prosocial behaviors at work. So, what is currently known about the prosocial-culture-work nexus? Studies of this nexus may be predicted to exist in the form of research on organization cultures of three types. One would be studies of specific organization cultures representing espoused Employer Value Propositions (EVPs). One would be etic studies using constructs of organization culture. The other would be emic studies, with the ‘thick description’ associated with ethnography/anthropology. An integrative literature review on the prosocial-organization culture-work nexus located 22 studies. Most studies are of the etic type, while others are mainly concerned with theory development. There is no evidence of a clear concept of organization culture being used in any study. The future research agenda for the prosocial-culture-work nexus follow from this. Constructs of organization culture need to be adopted and used. There is huge scope for EVP studies to explicitly and critically explore the breadth of the prosocial themes these often contain. Etic studies are the ones where the lack of organization culture constructs is most striking, given their methodologies. More emic studies with ethnographic/anthropological depth to explore, both work organizations in single countries, and in comparative studies across countries, are needed. Better clarified prosocial constructs will not in themselves advance knowledge if the organizational culture contexts in which prosociality exists remain under-appreciated.

## 1. Background: Introduction Why Organization Culture?

Culture encompasses all the human behaviors and values, norms and skills that are learned as conducive to survival in a given environment [1]. The interest and relevance of culture is the extent to which it provides, in contexts including work and organization, ‘soft power’ [2,3] that either aligns with ‘hard power’ and control, or even potentially replaces hard power and control. In work and employment, organization culture is the focus, with leadership and management expected to define and shape behaviors and values, norms and skills [4]. Organization culture is the way things are done, as well as the way things are understood, judged, valued, arranged, accomplished, talked about, and justified, in practice [5]. Organization culture, in this context, is a potentially all-encompassing and interconnected system [6], which has been critiqued precisely because it is so all-encompassing [7]. To operationalize organization culture and set boundaries, there have been more than 70 instruments developed [8], though, from among these several, some [9] are more widely used than others, such as the Competing Values Framework (CvF) [10,11]. There are also some popular models which are well-established and frequently used to help operationalize culture in the work organization context, such as Hofstede [12].

The CvF is a construct which describes and analyzes organization culture as having, as their core, one of four potential types; the compete type, the control type, the collaborate type and the create type. These culture types are operationalized with reference to the primacy in an organization of either doing things fast, doing things right, doing things together or doing things first. Leadership, management and front-line team culture will usually be shaped most by one of these, or some hybrid of two or even more organization culture types. The nature and salience of prosociality would be expected to vary according to the type of organization culture. Collaborate culture types would be expected to have significant prosociality in their purpose, leadership, management and teams.

Hofstede has a construct of culture which uses 6 dimensions [13] to map any national context, providing a template of a ‘typical’ member of a national culture which relates to workplace and organization interests and concerns. Awareness of culture type is expected to improve cross-cultural competence. The 6 dimensions are Power Distance, Individualism-Collectivism, Masculinity-Femininity, Uncertainty Avoidance, Long-term Short-term Orientation, and Indulgence-Restraint. Despite questions about the validity and value of the model, it has remained popular in work and management contexts for some decades, and continues to be referenced [14,15]. The potential alignment with prosociality is explicit and evident in some of these dimensions (individualism-collectivism), implicit in others (masculinity-femininity), and can be aligned with the other dimensions also.

There are three types of research that are available on organization culture which might be expected to appear in studies of the prosocial-work-culture nexus. One is research concerned with specific organizational cultures, and how the espoused Employer Value Propositions (EVPs) organizations articulate and promote [16,17,18,19] may or may not support prosociality. A second is research on organization members’ experiences at work, adopting etic approaches, measuring and exploring these with organization culture constructs, of which there are many, as well as prosociality constructs. A third is research adopting an emic approach, providing ‘thick’ descriptions of organization culture which are more ethnographic or anthropological in character, with prosociality a feature of those. These can take a broader view of work and relations in organizations, and explore the ‘low and ordinary’ experiences of people at work, in which power, identity and belonging are part of a culture in its entirety, and using forms of research engagement and researcher-informant relations, which accommodate roles including collaborators, apprentices, assistants and experts, as well as independent researchers [20].

‘Hyperculture’ and EVP studies [21] describe and analyze the explicit designs for culture manifest in the branding and other materials created as organizations actively define, espouse and manage their culture as a form of soft power. There are many and varied, local and global, annual lists of ‘best employers’ and ‘best places to work’, showcasing their EVPs. Organization culture is a feature of crowdsourced employer review web sources, such as Glassdoor [22]. In all these forms, organization culture materials are publicly available, either directly in the form of company materials which use symbols and language to convey their culture with, for example, explicit vision, mission, and value statements, or in review form on crowdsourced platforms. Organization culture research could capture the hyperculture and EVPs of organizational material in these forms, as cases or sets of cases, and analyzes those with reference to a theme, such as prosociality. Hyperculture and EVP research can be laudatory of positive and ideal role models, or critical of organizations seen to represent adverse types of culture, being toxic, declaimed and highlighted as in need of change.

The two other types of research in organization culture possible are defined here as the etic type of study and the emic type of study [23]. Etic approaches to organization culture research are analyst-centered, adopting operationalizations of constructs which are deemed to be relevant and valid, and are thought to be generalizable, regardless of the context of a study’s participant population, work sector, or place and nation [24]. Here, the aim is to capture culture member’s ideas and sentiments, the experience of what organization members tend to think and feel when accounting for ‘how things are done around here’. That may be done using instruments, surveys, or interviews. Studies may be comparative across organizations and countries, and may be an exploration of experiences using etic models of prosocial behavior, including, for example, constructs of OCB. It would be expected that these studies would include clear and explicit operationalizations of organization culture.

Emic organization culture research approaches are actor-centered, thickly described and context rich, reflecting ethnographic [25,26] and anthropological [27] perspectives and methods. They identify named and authentic actors and their own terms and experiences in a culture. They seek to systematize these in a ‘thick’ description of a specific culture’s language, stories, rituals and meanings. These studies can provide detail and nuance, with high ecological validity, and so generate knowledge with impact through the role-modelling of a culture type. The emic type of study often embodies, not only what can be seen as a hermeneutic rationale, but also an emancipatory purpose. That is to say, ‘cultural thinking’ and analysis is a way of having impact, of raising awareness and understanding of both the levers of change and constraints on change in organizations. Change with an emancipatory goal has often come to be voiced through culture analysis, whether concerned with local change in a specific organization or more global concerns around the big themes of diversity, human rights, freedoms and personal expression.

Each type of organization culture research has strengths and limitations. The limitation with hyperculture-type studies is that they tend to reify culture as a single, homogeneous system, downplaying the ways in which behaviors, values and norms are distributed psychologically and socially in any context. Etic research can be limited by being so nested in the validation of constructs and relations among them, to the point that it either becomes bewildering to keep track of all the relevant constructs or studies fragmented into specialized sub-zones within their own silos and self-referential communities. Emic studies may have high ecological validity which can be key to having impact as tangible implications emerge, but these are seen to be ‘merely descriptive’ and are not sufficient for theory-building and -testing. They provide stories and illustrations, which can enable powerful cases and comparison across cases, among individuals or organizations or countries, but those are vulnerable to becoming either redundant or questioned as events take their toll, and those once seen as ideals are tarnished or displaced.

Synthesis of the extant literature is challenging here, as it transpires that there is not much of substance to work with. There is not enough to generate a set of themes, either within or across EVP, etic and emic studies. Neither is there enough to substantiate a meta-analysis of quantitative studies and their findings, or to justify a metatheory. The focus is instead on a research agenda that flows from what has been found and what is missing, with provocative questions.

## 2. Method: Literature Review

This is an integrative review of a new and emerging topic [28], intending to be a holistic conceptualization and synthesis of the literature, and anticipating a synthesis around an initial or preliminary conceptualization of the topic of the prosocial-work-culture nexus, rather than a reconceptualization of existing models. If there had been sufficient literature, it might have enabled a Meta-analysis, but that has not been the case. Indeed, even a substantive review, critique and synthesis of the extant literature, weaving together ideas from the literature into themes, would prove challenging, given the extant literature found. The ultimate synthesis possible here is a research agenda and direction for future research on the prosocial-work-culture nexus.

**Figure 1:**
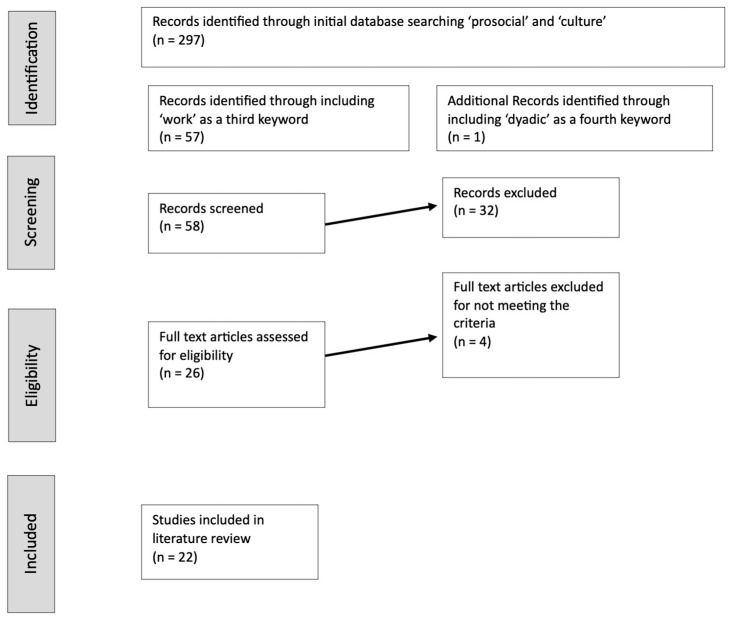
Literature Review Flow Chart.

EBSCO was used for the literature review, including the databases Health Source, APA PsycArticles, Psychology and Behavioral Science Collection, Socindex and Business Source Ultimate. A flow chart at Figure 1 shows the process. 

An initial search using the terms ‘prosocial’ and ‘culture’ generated 297 results. A review of these identified several clusters which were clearly not related to work contexts, being studies on young people, moral development and experimental studies using games. Rather than just excluding these, a second search, with the addition of the keyword ‘work’, generated 57 results. These abstracts were then reviewed for inclusion if they had, or seemed to have, a direct concern with prosocial behavior and work contexts. While doing that inclusion review, it was apparent that there was a lack of articles at a dyadic level. So, a further search on ‘dyadic’, ‘culture’, ‘prosocial’ and ‘work’ was done. This produced only 1 additional relevant source. The inclusion review resulted in a set of 26 full text articles which were reviewed. Of these, 4, when reviewed, were not directly or primarily about the prosocial-culture nexus in the organizational or work context. Thus, 22 articles were included in this review.

## 3. Results

The organization of the literature review is not temporal. However, culture seems to have hardly featured significantly as an element of studies of prosocial behavior at work before 2010 [29,30,31,32,33,34]. From 2010, culture as a construct has been more evident. That is perhaps coincident with culture being highlighted as a part of the field of prosociality in a text on prosociality [35]. For Mikulincer and Shaver, culture was a potentially significant force that might generate prosocial, generous and altruistic behavior, or block, inhibit or overpower prosocial behavior. Culture could be associated with, and be integral to, all levels of analysis, from theory building to mapping psychological processes and prosocial emotion in dyads. 

While there are more studies since 2010, these are not very extensive. There is not enough to generate a set of themes, either within or across EVP, etic and emic studies. There is no apparent dominant general theory, settled discourse on theory about the organization culture-work-prosocial nexus, or methodological paradigm. What has been done at the intersection of prosociality-work-culture are mainly etic studies, with an eclectic mix of studies concerned mainly with theorizing, and some on themes in leadership and HRM. Synthesis is challenging here as there is not much of substance to work with. There are not enough studies to enable and substantiate a meta-analysis of quantitative studies and their findings, or to justify a metatheory. Synthesis is instead proposed around a research agenda that flows from what has been found, and what is missing.

Etic type studies dominate, accounting for nearly 50% of those included. There was only one hyperculture/EVP-type study [36]. There were no emic type studies found. A variety of ‘other’ types of study were found. Some of these used specific theories, including a theory of an Internal Working Model of Others (IWMO); Self-Determination Theory (SDT); Lewinian force field theory and; Welzel’s theory of emancipation. In all, across all 22 full text articles reviewed, there were only three specific mentions of an organization culture construct. Two studies mentioned Hofstede, and one mentioned Cameron and Quinn. The studies that cited Hofstede did not actually use his model, and the study citing Cameron and Quinn only used a limited part of that model, not the whole model.

In the etic studies, using a variety of established instruments and measures, there was little consistency with the prosocial and work-related constructs they did use [37,38,39,40,41,42]. Organization Citizenship Behavior (OCB) was cited twice. Human Resource Management (HRM) was also cited twice. In two articles, an element of leadership theory was significant, and constructs around that were mentioned. 

Of the other studies that were found, a synthesis of theory of some kind was a common feature [43,44,45,46,47,48,49,50,51,52,53]. One was concerned with a synthesis around an integration of Behaviorism, Humanism and Positive Psychology. One was concerned with Confucian and Aristotelian concepts. Others dealt with themes across a range of general motivation and reward theories; socialization and semiotics; socialization theory/national business ideology; cooperation and competition; deviance; social preferences; altruism; meaningfulness; helpfulness; agentic motivation; well-being. 

In short, there were significant differences between what was expected in the literature and what was found. There was very limited reference, virtually none, to any theory or construct of culture or organization culture in studies of the prosocial-culture-work nexus. There was much consideration of theory and less empirical research of any type. There were no emic studies of the prosocial-culture-work nexus. 

Table 1 shows a summary of all 22 studies included. The first columns indicate the type of study using the expected types (EVP, Etic, Emic) and the emergent ‘other’ themes found, also indicating those that are primarily theoretical, and whether the study is either a single country or comparative study. The other columns show author, year, key findings and the prosocial-culture-work nexus. These are also categorized as having data on either ‘One Country’, ‘Comparative’, or as being ‘Theory’ oriented.

## 4. Conclusions

The literature review did not produce the expected range and mix of studies representing EVP, etic and emic studies to enable an integrative review and synthesis of the prosocial-organization culture-work nexus. Most tellingly, there was hardly any reference at all to any constructs of organization culture in any type of study. This is rather remarkable. Alongside construct clarity, there is scope for culture to be a unifying theme to connect the diverse behavior-oriented and societal concerns around prosociality in work. For that, the prosocial-organization culture-work nexus needs to be a domain in which a variety of types of research are better represented, for knowledge and evidence to be both more robust and have impact.

What that means is synthesized around a future research agenda, with regard to the research themes and questions which might be associated with the prosocial-work-culture nexus, and the methodological options of hypercultures/EVP, etic and emic studies; and also, how the themes of theory, leadership and HRM might also be dealt with, either in their own right, or within those methodological options. There is a need for more EVP/hyperculture types of studies. There is a need for greater coherence in etic studies, with use of models like the CvF framework. More emic type studies with ethnographic/anthropological data, both in one country and in comparative studies, are needed. There is scope for more criticality in the use of culture as a construct in the work-prosocial context. One significant concern that all this raises, in the context of this special edition, is that the endeavor to more definitively isolate prosociality as a distinctive construct may risk cutting it off from the narratives and dynamics of work and culture as they exist around all kinds of ‘social’ tagged themes. What that means is touched upon. More studies of each type that explicitly adopt and work with constructs of organization culture are needed to deliver impact.

There is a need for more EVP/hyperculture types of studies. It was surprising to find so few studies of the EVP/hyperculture type. To recap, EVP/hyperculture studies are those which examine the explicit and espoused cultures which work organizations consciously design, and represent to others how the organization is a great place to work, including being prosocial. The extent to which ideal types of prosocial EVPs are integral to the ways that the ‘best’ employers represent themselves is an inescapable context for understanding and exploring prosociality. Prosociality, however inclusively or narrowly defined, is usually a tangible part of the managed culture of an organization, not something that emerges solely from interpersonal interactions among pairs, teams, and across hierarchies. In the organization and work context, the most direct, simple and explicit form of culture analysis is that of the hyperculture or EVP case [52], and the highly visible lists of best employers and best places to work. Perhaps because there is a ‘hypercultural’ and pragmatic purpose to this, to help leaders manage culture, researchers are less interested in exploring the prosocial, as it appears in these. Even so, the appreciation and interpretation of ‘hyperculture’—consciously designed and managed cultures—and prosociality themes is still potentially considerable. It is not necessary to have resolved debates about whether organization culture can ever be managed, beyond the superficial prescription of familiar and common values, to see that even some simple content analysis of EVPs might help situate the examination of prosociality in work organization contexts.

Studies using etic approaches, for example, using the CvF construct of organization culture types, need more coherence in operationalizing culture and the contextualization of that in the focus and findings of studies. Research contextualizing prosociality in EVPs and organization hypercultures is needed, alongside studies concerned with leadership and HRM. This can be seen in some research on the interpersonal dynamics that enable and constrain proactivity, persistence, performance and productivity, exploring aspects of competition, prosocial motivation and collaborations [53]. While a construct of organization culture is not explicitly used by Grant and Shandell, the themes of competition and collaboration mirror exactly those of two core organization culture types in the CvF framework, those of compete and collaborate culture types. The other types in the CvF model, ‘create ‘and ‘control’ culture types, are missing from the analysis. The interest in the nature of rivalries and friendships as potentially double-edged swords in prosociality in organization cultures combining competition and collaboration is highlighted. That a balance of competing and contributing will set the challenges facing some leaders, managers and front-line employees in teams is also highlighted. However, the cultural and practical themes of creativity and control matter in some leadership, management and front-line employee team contexts. The thematic analysis of organization culture can be a way of framing the prosocial to stretch and include those kinds of themes too, and avoid becoming trapped in a limited perspective on balancing competition and collaboration, for, creativity and control need to be considered too. In this way, borrowing from and using etic models from the domain of organization culture can contribute to framing and focusing research questions and themes of salience to exploring prosociality.

The nature and salience of prosociality would be expected to vary according to the type of organization culture an etic perspective operationalizes. Collaborate culture types would be expected to have significant prosociality in their purpose, leadership, management and teams. As ‘collaborate’ culture types are perceived to be appealing, they can be an integral feature of EVPs; most employers wish to present themselves as having collaborate culture characteristics. Whether this is delivered or not is clearly one theme and question. How prosociality is, or is not, featured in leadership, management and front-line teams in control, compete and create culture types provides another set of themes and questions.

More emic type studies with ethnographic/anthropological data, both in one country and in comparative studies, are needed. A central theme and question here can be the validity and consequence of what has been discussed around ‘WEIRD’ culture [54,55]. WEIRD is an acronym for Western, Educated, Industrialized, Rich and Democratic. The twist in the name is of course that far from being ‘weird’, in the sense of being unusual, different and ostracized, the WEIRD are now common, dominant, and to be emulated. Yet, the culture of the WEIRD is distinctive, being highly individualistic, self-obsessed, control-oriented, nonconformist and analytical. In short, not prosocial. It is a culture where people focus on themselves over relationships and set roles. The acronym WEIRD is a striking and potentially innovative way of reframing the traditional ‘individualistic versus collectivist’ contrast across cultures under the labels of ‘Western’ and ‘non-Western’. In the limited literature located, which explicitly explores prosociality at work, this features in the questioning of the ascendancy and of the individualistic and Western being challenged by the economic power of the East, especially China [56]. As well, it features alongside more assertive post-colonial influences in contexts such as Africa [57] and the Islamic world, concerned with evidencing and practicing effective organization, leadership and management, which respect and embody local heritage such as ‘shemswian’ companionate leadership. It is always necessary to highlight that heterogeneity within cultures, and therefore contrasts among them, can be hugely overstated and result in unhelpful, ideologically oriented, zero-sum thinking and conflict about good and bad cultures. One of the potential contributions of more emic studies is their capacity to avoid presenting false heterogeneity, or generating or sustaining unhelpful division and conflict in the exploration of themes such as prosociality. Thick descriptions of real lives in work contexts may lack in the validity and generalizability of etic types of study, but they can humanize and personalize the subject and its exploration. 

Criticality can be achieved in various ways around organization EVP/hyperculture research. Organization culture contexts can be understood to be of three potential types: integrated, differentiated, or fragmented [58]. Integrated hypercultures and EVPs exist when there is wide consensus on the basic beliefs and appropriateness of behaviors within the organization. Differentiated hypercultures and EVPs exist when multiple groups within an organization possess diverse and often incompatible views and norms; there are subcultures, and conflict among these is expected. Most complex organizations have subcultures, professional groups, managers, staff groups and countercultures. These may be powerful catalysts for innovation and improvement, or defenders of the status quo. Differentiated hypercultures and EVPs may diverge to the point that they become fragmented. Fragmentation exists where cross-organizational consensus and norms are absent, and an organization can be characterized by shifting alliances and allegiances, with considerable uncertainty and ambiguity, and there is open conflict around behaviors, values, norms and skills. 

Themes in theory that seem worth further exploration are: using explicit constructs of culture, including personal-cultural and collective-cultural perspectives, the legacy of altruism, caring as a focus of advocacy and campaigning, variations in businesses being socially oriented, types of prosocial motives, knowledge and teaching in business ethics and organization studies.

In reviewing these themes and questions reflecting on the potential of culture as a context within which prosociality might be better operationalized and studied another challenge occurs. This is that the endeavor to more definitively isolate prosociality as a distinctive construct may risk cutting it off from the narratives and dynamics of work and culture as they exist around all kinds of ‘social’ tagged themes. A list of just some of those social tagged themes would include:

- Social Justice
- Social Impact
- Social Innovation
- Social Capital
- Social Responsibility
- Social Investment
- Social Enterprise
- Social Good

The purpose of, and interest in, prosociality should not become confined to a single construct set apart, but be aligned and connected more broadly with the range of narratives around the ‘social’ and what ‘prosocial’ cultures themes are salient to, in the contexts of social justice, social capital, social impact, social innovation and so on. In other words, culture as a perspective suggests that it is a mistake to try to isolate and operationalize within ‘prosociality’ everything that pertains to broader themes of justice, capital, impact, innovation and so on. The prosocial-culture-work nexus is a dynamic touching on several significant domains of social policy and social practice, which may be included and appreciated in studies of any type, either EVP, etic or emic. 

The limitations here include the choice of only one search tool, the set of keywords used, and the search strategy used. The focus was specifically on the keywords of prosocial, culture and work in one search engine only. It would be possible, with more resources, to widen the range of keywords (‘kindness’, ‘compassion’ and so on), search tools and search strategies used, to complete a more systematic literature review. That might also include a strategy of targeting more journals with business, management, HRM, organization, ethics and international/cross-cultural remits. Also, specialist journals in the emic domain, concerned with ethnography and anthropology, could be included. It would be expected that the amount of material located would increase, though the underlying patterns in what might be found might also be expected to be similar.

More studies of each type that explicitly adopt and work with constructs of organization culture are needed to deliver impact. There is a duality in organization of hard power and soft power, control and culture. On the one hand, the prosocial-organization culture-work nexus can be aligned with leadership and HRM seeking to enable and tap into soft power through high trust, self-regulation, situational agility and shared purpose seems aligned with prosociality; on the other hand, leadership and HRM grounded more in the use surveillance, systems and strong leading may lead to non-prosociality. Who may benefit from greater prosociality at work, and who may be threatened? In the work and employment context, the potential impacts and beneficiaries that matter most are typically around attracting and retaining the right people, managing for high performance, and achieving equality, diversity and inclusion. Simply clarifying prosocial constructs cannot, in itself, enable impact; contextualizing the prosocial in the organization culture-work nexus can.

Alongside prosocial construct clarity, there is scope for other constructs, in this case, organization culture, to be adopted if they are to have some significance and potential as a unifying theme to connect the diverse behavior-oriented and societal concerns around prosociality in work [59,60]. Might interest in and exploration of the prosocial represent a form of timely correction to ‘WEIRD’ individualism and neoliberal self-expression [61]. Or, might it be part of a soft power shift, recognizing forms of traditional collectivism and collectivist thinking [62], in which the promise of greater kindness, compassion and so on may come for some, but at a price for others in how the ‘non-WEIRD’ can prioritize conformity under forms of leadership which are exceptionally repressive? Prosociality, as it interacts with work and culture, may be at the heart, not only of kinder experiences in workplaces; it may be a key site in which the journey of cultural modernization shifts from survival and traditional cultures to self-expression and rational-secular cultures.

We are left with the following key questions:

1. For EVP and etic studies, which models of organization culture provide the best means of capturing and exploring prosocial phenomenon at work in a useful and critical way?
2. For emic studies, what insights from operationalization of prosociality can be taken into work settings in which they can alert and sensitize the ethnographic/anthropologically minded researcher, whichever methods are being used?
3. Is it necessary (or even possible) to first synthesis and develop theory around the prosocial-work-culture nexus to avoid being entangled in a proliferation of diverse theories being adopted and used in empirical studies?
4. Is the lack of coverage of large cultural domains and the predominance of WEIRD contexts for prosocial-work-culture studies a significant problem or a straightforward opportunity?

More studies of any and all kinds which explicitly use a construct and appreciation of organization culture in the prosocial-work nexus can only help. They can help to contextualize the clarification of meanings and operationalizations of prosociality with some of the great challenges being encountered, both locally in myriad work relationships, and globally across a multi-polar world. That is a world in which soft powers, both old and new, could be searching for and finding ways in which prosociality enables constructive co-existence more than it divides or differentiates.

## Figures and Tables

**Table 1:** Literature Review Studies.

Type of Study		Authors	Year	Key Findings	Conceptual and/or Empirical
ETIC	Theory; Social psychology	[29]	1986	13 specific forms of prosocial behavior are described.	Conceptual; Behaviors vary according to whether they are functional or dysfunctional, role prescribed or not prescribed extra-role, and directed toward an individual or organizational target.
ETIC	Theory; Comparative	[31]	2000	Prosocial behaviors play a significant role in developing countries.	Conceptual; To appreciate specifically what might be done to promote volunteering and OCB in sub-Saharan Africa, as an example of a developing country context.
ETIC	Theory; Investment	[33]	2008	Socially responsible investment (SRI) strongly resembles pro-social behavior.	Conceptual; SRI creates opportunities for businesses to thrive relative to their competitors, reflecting complex combinations of psychological predispositions and institutional and cultural processes.
ETIC/Comparative;	Comparative: European	[37]	2011	National business ideology and employees’ prosocial values. Organization Culture construct-Hofstede referenced.	Empirical; Data from 17 European countries; European Social Survey; GLOBE Country scores; International Social Survey Program (ISSP). World Bank’s corporate corruption index.
ETIC	One Country: Taiwan	[38]	2015	Employees’ organizational citizenship behaviors (OCBs).	Empirical; Chinese employee–supervisor dyads from Taiwan in 13 financial institutions. Impression management motives may undermine the positive effects of prosocial values.
ETIC	Comparative: European	[39]	2019	Understanding of how, and in which cultural contexts, formalization creates value for organizations.	Empirical; Data from 7537 employees in 267 organizations across 17 countries. From own research team. Included five formalization items from the Competing Value Framework (Cameron & Quinn Org Culture construct referenced). Genuinely cross-cultural and global team and survey, but confused on focus and culture.
ETIC	One Country: Netherlands	[40]	2020	Highly engaged relations with coworkers and low engaged coworker.	Empirical; Dyads of coworkers. 254 Various sectors, mainly health and education.
ETIC	One Country: India	[41]	2021	Mediating role of job efficacy.	Empirical; Nurses’ perception about HRM system and prosocial organizational behavior. Non-profit hospitals in India. 387 nurses.
ETIC	One Country: USA	[42]	2021	Develop an instrument for measuring pro-social rule breaking (PSRB).	Empirical; University employees. Young, white, female employed students. Three studies with contextual performance ratings made by supervisors and coworkers. Negative impact on perceptions of supervisors.
ETIC	One Country: Netherlands	[34]	2009	SCOOM Hypotheses. Interventions geared toward job enrichment and team-based working.	Empirical; Three panels of supervisors and panel of supervisors/employees in healthcare and other sectors.
Other/Semiotics	Theory; Semiotics	[32]	2007	Interplay between personal-cultural and collective-cultural valuation of different forms of conduct.	Conceptual; ‘Prosocial’ acts may become semiotically transformed into ‘antisocial’ ones, and vice versa.
Other/Organization Case	One Country: UK	[43]	2012	The legacy of altruism in healthcare.	Empirical; 83 interviews part of a broader study. Promoting altruism in the context of healthcare is contradictory and misguided. Instead, an approach to clinical care that is prosocial and empathic is recommended.
Other/Theory	One Country: USA	[44]	2021	Actively Caring for People (AC4P) Movement.	Conceptual; Integration of Behaviorism, Humanism and Positive Psychology ‘throughout the world’.
Other/ Enterprise	Comparative	[45]	2019	Why some businesses are more socially oriented than others in their policies and activities.	Empirical; Entrepreneurs in 43 countries.
Other/Collectivism	Theory	[46]	2022	Four different types of prosocial motives.	Conceptual; Collectivism in non-Western cultures.
Other/Disciplines	Theory	[47]	2014	Business Ethics and Organization Studies.	Conceptual; Performing and providing meaningful work beyond national economic growth alone, striving for meaningful lives.
Other/Philosophical	Theory	[48]	2022	Four different types of prosocial motives.	Conceptual; Aristotelian versus Galilean thinking. Orchestration and bringing together prosocial motives of different kinds.
Other/Leadership	One Country: USA	[49]	2016	Develop and validate the Virtuous Leadership Questionnaire (VLQ).	Conceptual; Model of Confucian vs Aristotelian ‘thinking’ as dominating Western and Eastern thinking. MBA students and follower-leader pairs.
Other/Leadership	Theory	[50]	2018	Ethical leadership interacts with co-worker ethicality. The interactive effect. But, mainly leaders shape behavior.	Empirical; Canadian Military. Importance of leaders and co-workers in shaping the behavior of organizational members.
Other/HRM	One Country: USA	[30]	1995	Explore a new pay plan. Roles of Workplace Justice, Achievement Striving, and Pay Satisfaction.	Empirical; case of Consumer Products company. No company details.
Other HRM	One Country: England	[51]	2013	PSOB exhibited by National Health Service employees.	Empirical; Value-driven HR may offer one means of maintaining and encouraging both altruistic and conscientious act of PSOB.
EVP/Case Study	One Country: USA	[36]	2022	Describe a role-model organization-helping culture.	Conceptual; Role model ‘helpful leader’, norms of reciprocity, reinforcement of norms, willingness to leave slack in employees’ schedules.
